# Effect of heat input on microstructure, wear and friction behavior of (wt.-%) 50FeCrC-20FeW-30FeB coating on AISI 1020 produced by using PTA welding

**DOI:** 10.1371/journal.pone.0190243

**Published:** 2018-01-11

**Authors:** Cihan Özel, Turan Gürgenç

**Affiliations:** 1 Department of Mechanical Engineering, Firat University, Engineering Faculty, Elazig, Turkey; 2 Department of Automotive Engineering, Firat University, Technology Faculty, Elazig, Turkey; Beihang University, CHINA

## Abstract

In this study, AISI 1020 steel surface was coated in different heat inputs with (wt.-%) 50FeCrC-20FeW-30FeB powder mixture by using plasma transferred arc (PTA) welding method. The microstructure of the coated samples were investigated by using optical microscope (OM), scanning electron microscope (SEM), X-ray diffraction (XRD) and energy dispersive X-ray (EDS). The hardness was measured with micro hardness test device. The dry sliding wear and friction coefficient properties were determined using a block-on-disk type wear test device. Wear tests were performed at 19.62 N, 39.24 N, 58.86 N load and the sliding distance of 900 m. The results were shown that different microstructures formed due to the heat input change. The highest average micro hardness value was measured at 1217 HV on sample coated with low heat input. It was determined that the wear resistance decreased with increasing heat input.

## Introduction

The wear is very important for performance and service life of metallic machine parts [1]. The protection of metallic machine parts against wear has significance for economic approach [2]. Surface coating is one of the most useful and economic method for protect machine parts against wear [3, 4]. Surface coating is generally used industries, such as agricultural, mining and soil [5]. Plasma transferred arc (PTA) welding plays important role on the surface coating technology. PTA welding method has some advantages such as high energy density, excellent arc stability, high deposition rate, high welding speeds, lower heat input, low thermal distortion of the parts and low cost equipment [6–8].

Fe-Cr-C coating is known good wear and corrosion resistance by the formation of M_7_C_3_ and M_23_C_6_ carbides [9, 10]. Fe-Cr-B-C and Fe-Cr-W-B-C coatings are known as wear resistance coatings [11, 12]. The influence of heat input on microstructure and wear were investigated in many studies [13, 14]. Also in some studies it was investigated that the molecular dynamics of coating process [15, 16].

In the literature no more studies Fe-Cr-W-B-C coating on AISI 1020 steel by using PTA welding method was encountered. In this research, Fe-Cr-W-B-C coating with high content of FeB and FeW were coated on low carbon steel surface by using PTA welding method. After the microstructure of the coatings had been analyzed by the OM, SEM, EDS and XRD methods, the microhardness, wear and friction coefficient properties of the coating layers were determined. Finally the worn surfaces were investigated by using SEM microscope.

## Experimental details

**Fig 1:**
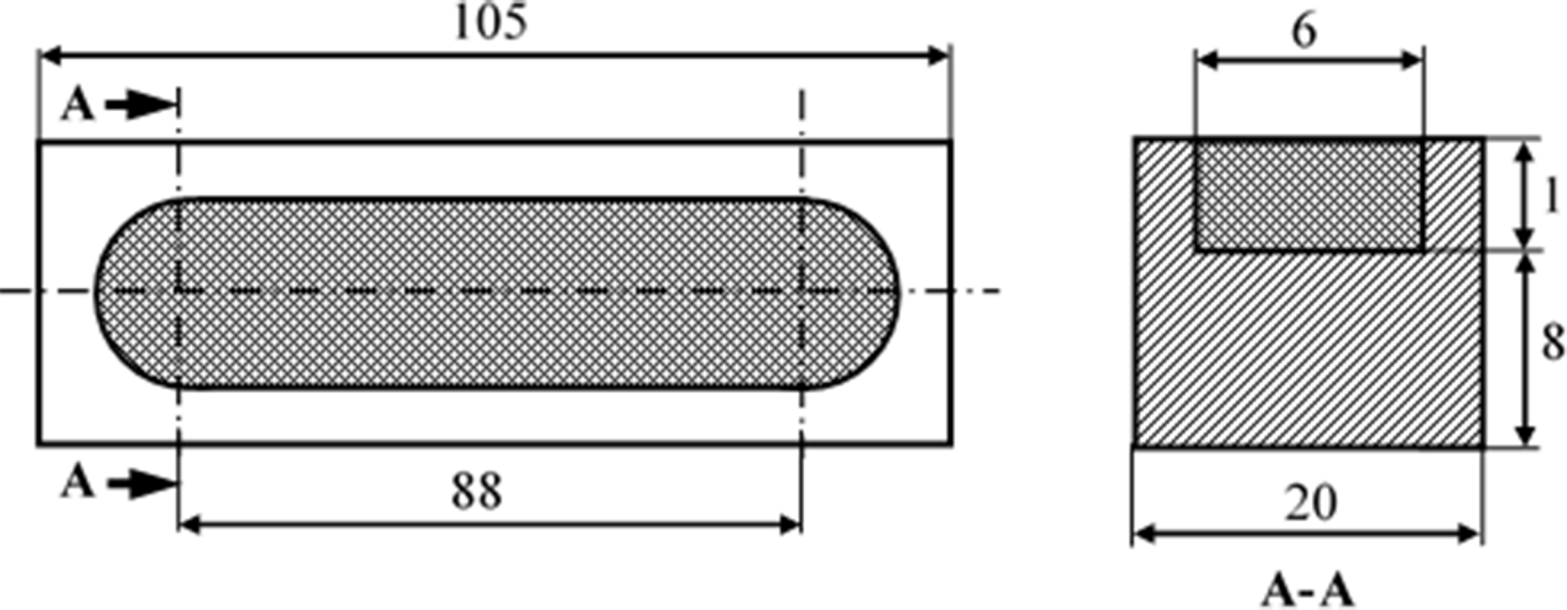
Dimensions of the substrate material (mm).

In this study, commercially buying AISI 1020 steel was used as substrate material and prepared dimensions in CNC milling machine which were given in Fig 1. For surface alloying materials FeCrC (buying from Eti Krom co inc.Turkey), FeB (buying from AVEKS AS) and FeW (buying from AVEKS AS) were used and ferro alloys were grinding approximately 38 μm size. The chemical compositions of AISI 1020 steel and ferro alloys were given in Table 1.

**Table 1:** Chemical compositions of materials (wt.-%).

Material	Cr	B	C	P	Si	W	Mn	Fe
**AISI 1020**	-	-	0.20	0.02	0.286	-	0.356	Bal.
**FeCrC**	66.77	-	7.95	0.007	0.550	-	-	Bal.
**FeW**	-	-	0.045	0.05	0.520	79.26	-	Bal.
**FeB**	-	18.22	0.30	0.05	0.500	-	-	Bal.

The surface of the substrate material was cleaned with acetone before coating and dried in the furnace at 60°C for 30 minutes. The ferro alloy powders were dried in the furnace at 110°C for 1 hour to remove the moisture. These powders containing wt.-% 50FeCrC-20FeW-30FeB, were weighed with a precision scale and this mixture was stirred for 1 hour at 150 rpm/min in a mechanical stirrer and placed in to the channel with alcohol. Samples were placed in the furnace and kept there at 100°C for 1 hour to remove the moisture. After the samples had been removed from the furnace, they were kept until the room temperature was reached and the surface coating processes were done by using the Thermal Dynamics WC100B brand PTA welding device with the parameters given in Table 2. After the coating process the samples were put on to cool at room temperature. Other details of experiments were given in our previous study [17].

**Table 2:** Coating parameters.

Sample	S1	S2	S3	S4
Current (A)	140	160	140	160
Coating speed (m/min)	0.15	0.15	0.1	0.1
Voltage (V)	19–20			
Plasma gas (Argon) flow rate (l/min)	0.5			
Shielding gas (Argon) flow rate (l/min)	8			
Coating length (mm)	2			
Heat input (kj/mm)	0.585	0.704	0.878	1.056

The wear rates were found by using Eq 1. During the wear test the change in friction force dependent on the sliding distance was recorded and transferred to the computer via a data logger. Finally, the worn coating surfaces were analyzed using a SEM microscope.

$$Wear\;rate=(m_{f}-m_{l})/m_{f}$$ (1)

where *m*_*f*_ (g) is the initial mass of the sample and *m*_*l*_ (g) is the mass after wear.

## Results and discussion

### Microstructure

**Fig 2:**
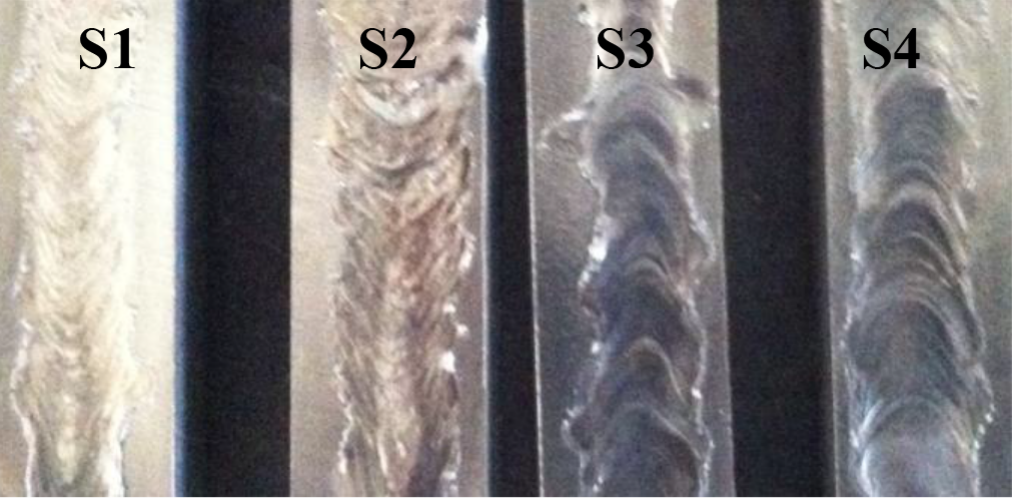
Macro photographs of coating surfaces.

The macro photographs of the coating surfaces was shown in Fig 2. It could be seen that there is no crack or porosity on the coated surfaces and that the surface is modified with the melting of Fe-Cr-W-B-C reinforcing powder mixture with the substrate material. In Table 3 some coating properties were given. As it seen coating layers depths and interface regions heights increased with increasing heat input.

**Table 3:** Coating properties.

Sample	H (μm)	h (μm)
**S1**	1440	2.24
**S2**	1510	8.02
**S3**	1610	13.06
**S4**	1690	14.26

**H:** Coating maximum depth; **h**: Average interface height taken from five different points

**Fig 3:**
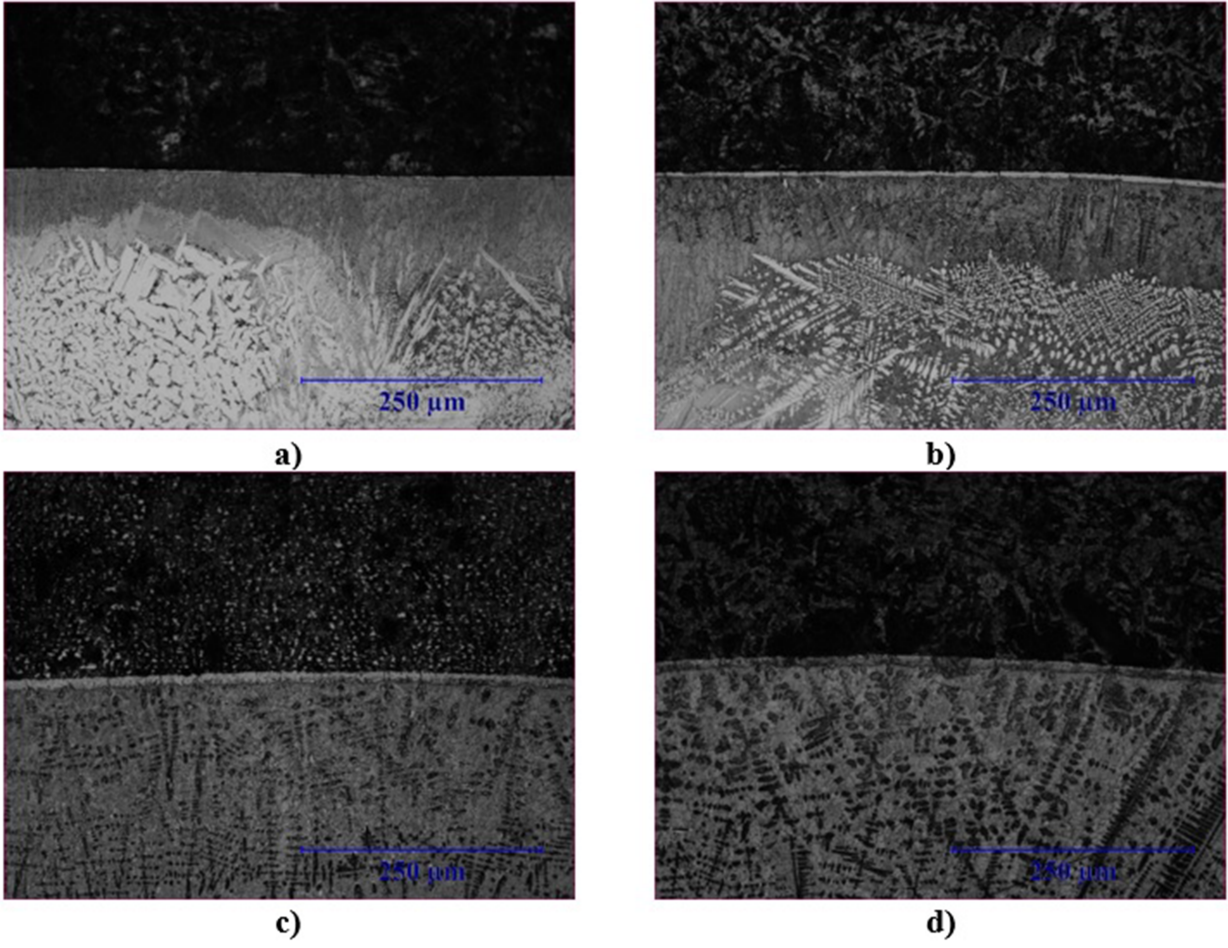
OM photographs of coating layers (x200).

The OM photographs of the coating layers were shown in Fig 3. As it seen there are no cracks, porosity, and gaps between AISI 1020 and the coating region. As it seen, different microstructures appeared in the coatings with the change of heat input. This can be explained by the change of the melting amount of the substrate material with the change of heat input and the chemical composition of the coating. Coating layer of S1 consists of mainly borides and hexagonal and strip-shaped carbides (Fig 3A). Also the amounts of carbides and borides in the coating layer are high. S2’s coating layer consists of mainly fine dendrites and eutectics (Fig 3B). Because of increasing melting density of substrate material with the increased heat input and the rapid cooling, S3 and S4’s coating layers consist of dendrites and interdendritic eutectics.

**Fig 4:**
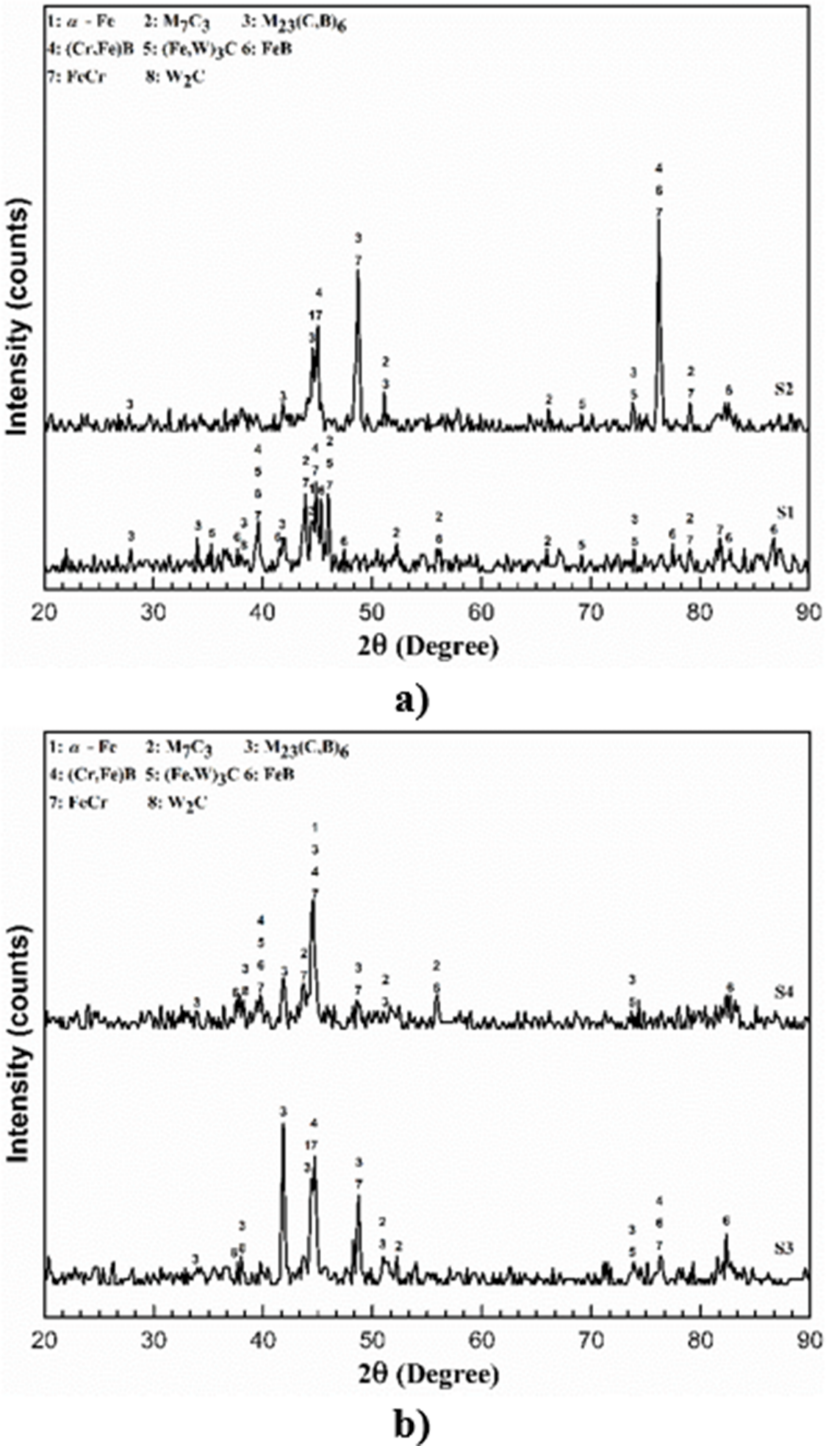
XRD results.

XRD analysis results of coated samples were shown in Fig 4. According to the XRD results, all coating layers mainly compose of M_23_(C, B)_6_ (M = Cr, Fe, W) carbide, M_7_C_3_ (M = Cr, Fe, W) carbide, (Cr, Fe)B boride, FeB boride and FeCr. As can be seen from the XRD analysis results The size intensity and form of phases in the coating layers vary depending on the changing heat input and different solidification times [18].

**Fig 5:**
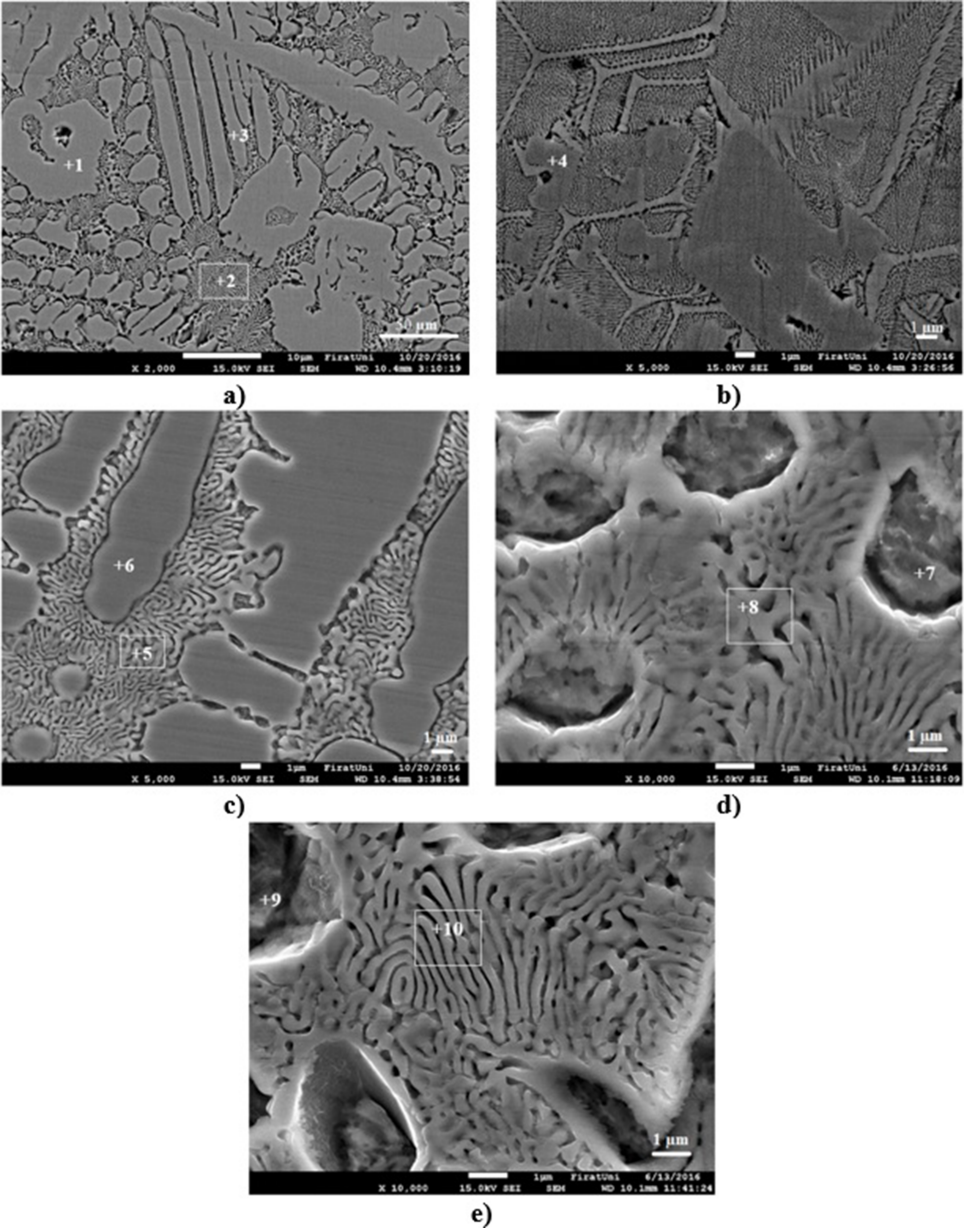
**SEM photographs of coating layers.** a) S1 (x2000), b) S1 (x5000), c) S2 (x5000), d) S3 (x10000) and e) S4 (x10000).

The SEM photographs of coating layers were shown in Fig 5. EDS results taken from point 1 (Fig 5A) which contains wt.-% 30.20C-25Cr-39.67Fe-5.13W elements. According to the Cr/Fe ratio this phase was thought to be M_23_(C,B)_6_ (M = Cr, Fe, W) carbide [19, 20]. Eutectic phase (Fig 5A-point 2) contains wt.-% 29.89C-12.25Cr-48.36Fe-9.49W elements and strip-shaped carbide (Fig 5A-point 3) contains wt.-% 31.40C-15.95Cr-46.26Fe-6.39W elements. The phase in Fig 5B point 4 is boride and it contains wt.-% 31.16C-24.63Cr-44.22Fe elements. According to the EDS results this boride was thought to be (Cr, Fe)B boride. Eutectic phase (Fig 5C-point 5) contains wt.-% 17.16C-13.51Cr-61.43Fe-7.9W elements and dendritic phase (Fig 5C-point 6) contains wt.-% 23.55C-15.74Cr-55.46Fe-5.25W. EDS results taken from point 7 and 9 the dendrites contains wt.-% 19.00C-6.41Cr-71.74Fe-2.86W and wt.-% 16.23C-6.40Cr-73.88Fe-3.49W elements respectively. Eutectic phases in Fig 5D-point 8 consists of wt.-% 14.16–15.12Cr-63.43Fe-7.29W and Fig 5E-point10 consists of wt.-% 16.65C-13.02Cr-64.41Fe-5.92W elements. It was concluded that an increase in heat input eutectic phases leads to increased amount of Fe elements.

### Microhardness

**Fig 6:**
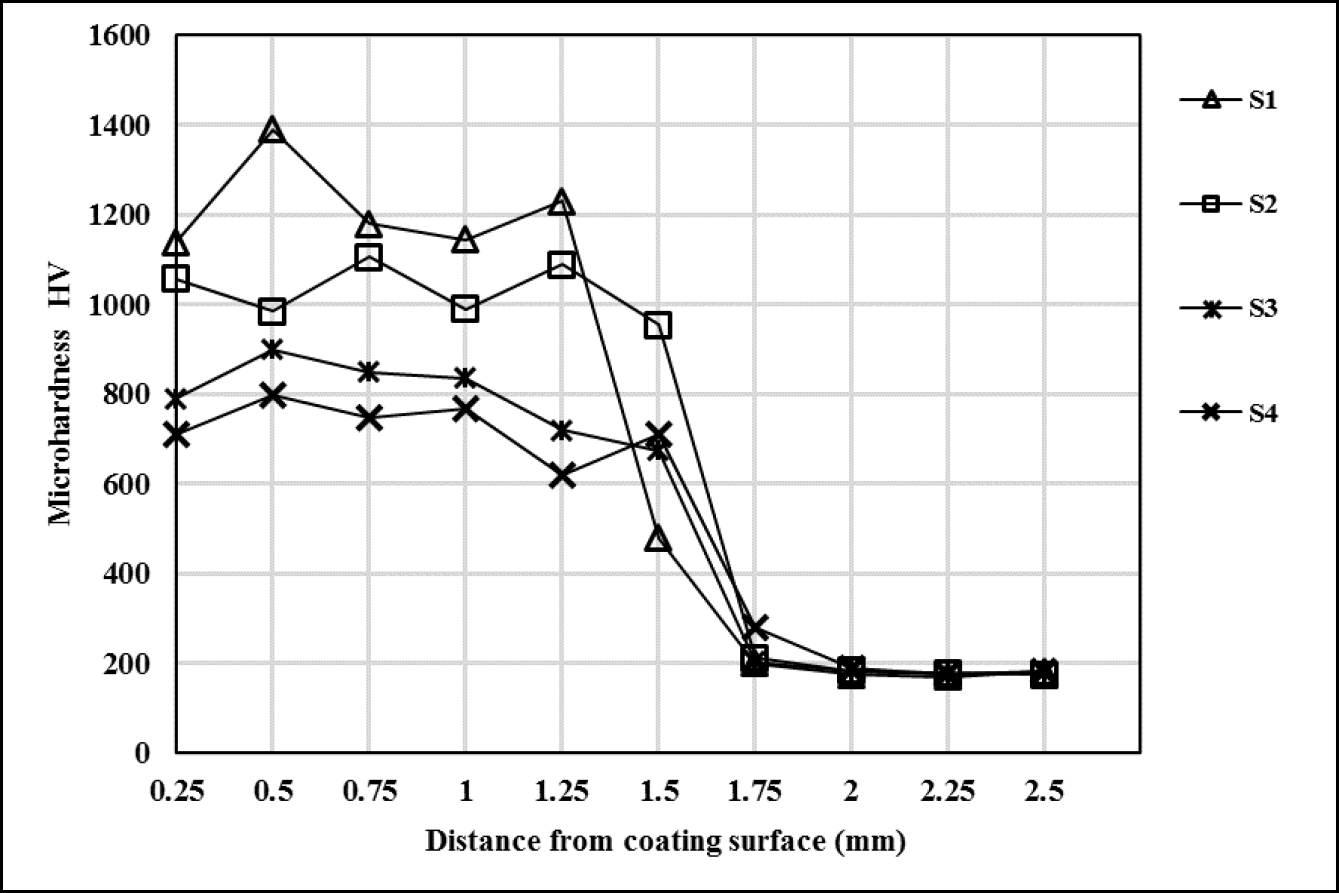
Microhardness distribution of coating layers.

The average and maximum microhardness of the coating layers were given in Table 4. The coated samples microhardness are higher than the AISI 1020 due to the hard carbide and boride phases. Coating layers average microhardness were changed between 725–1217 HV and decreased with increasing heat input. Maximum microhardness were measured at 1390 HV in S1 which is coated with lowest heat input. The microhardness distribution of coating layers were seen in Fig 6. The dendritic coating layers microhardness changed insignificantly from top surface to substrate.

**Table 4:** Average and maximum microhardness of coating layers.

Sample	V	A	Q	HV_(avg.)_	HV_(max.)_
S1	0.15 m/min.	140 A	0.585 kj/mm	1217 HV	1390 HV
S2	0.15 m/min.	160 A	0.704 kj/mm	1030 HV	1107 HV
S3	0.1 m/min.	140 A	0.878 kj/mm	795 HV	899 HV
S4	0.1 m/min.	160 A	1.056 kj/mm	725 HV	797 HV

**V:** Coating speed (m/min.); **A:** Current (A); **Q:** Heat input (kj/mm); **HV**_**(avg.)**_: Average microhardness of coating layer; **HV**_**(max.)**_: Maximum microhardness.

### Friction and wear

**Fig 7:**
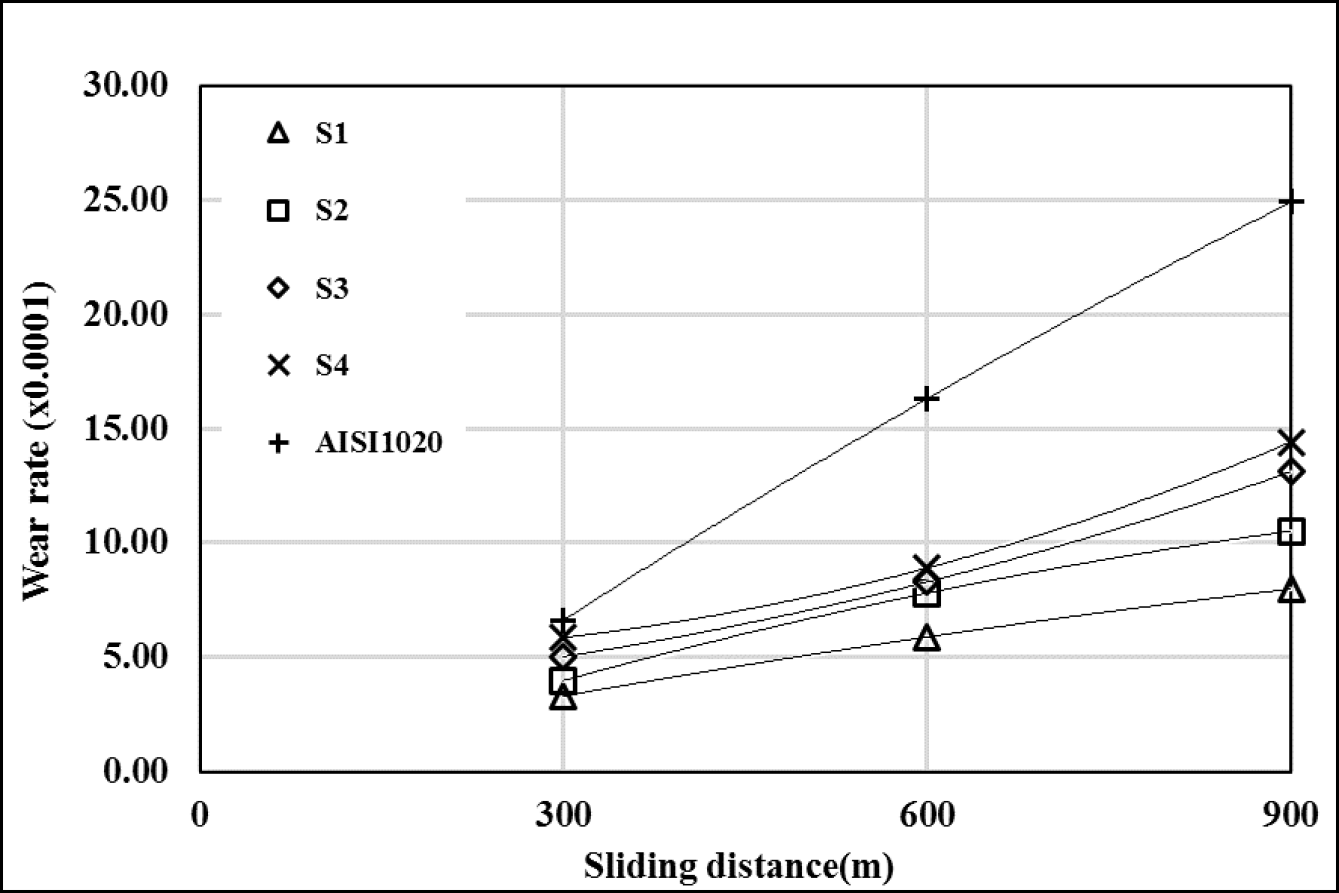
Wear rates at 19.62 N load. (The wear rate is found by multiplying the value of x-axis by 0.0001).

**Fig 8:**
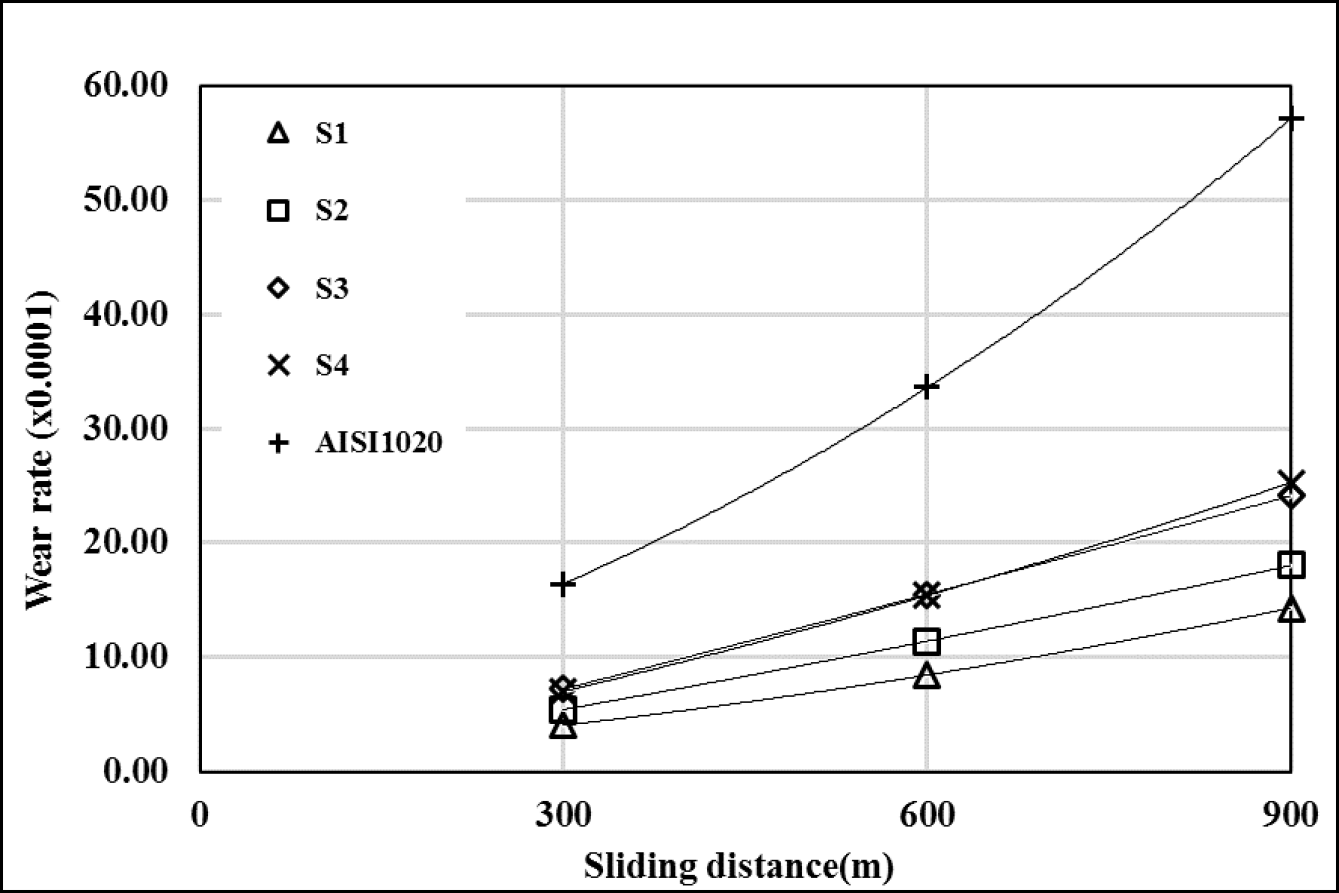
Wear rates at 39.24 N load. (The wear rate is found by multiplying the value of x-axis by 0.0001).

**Fig 9:**
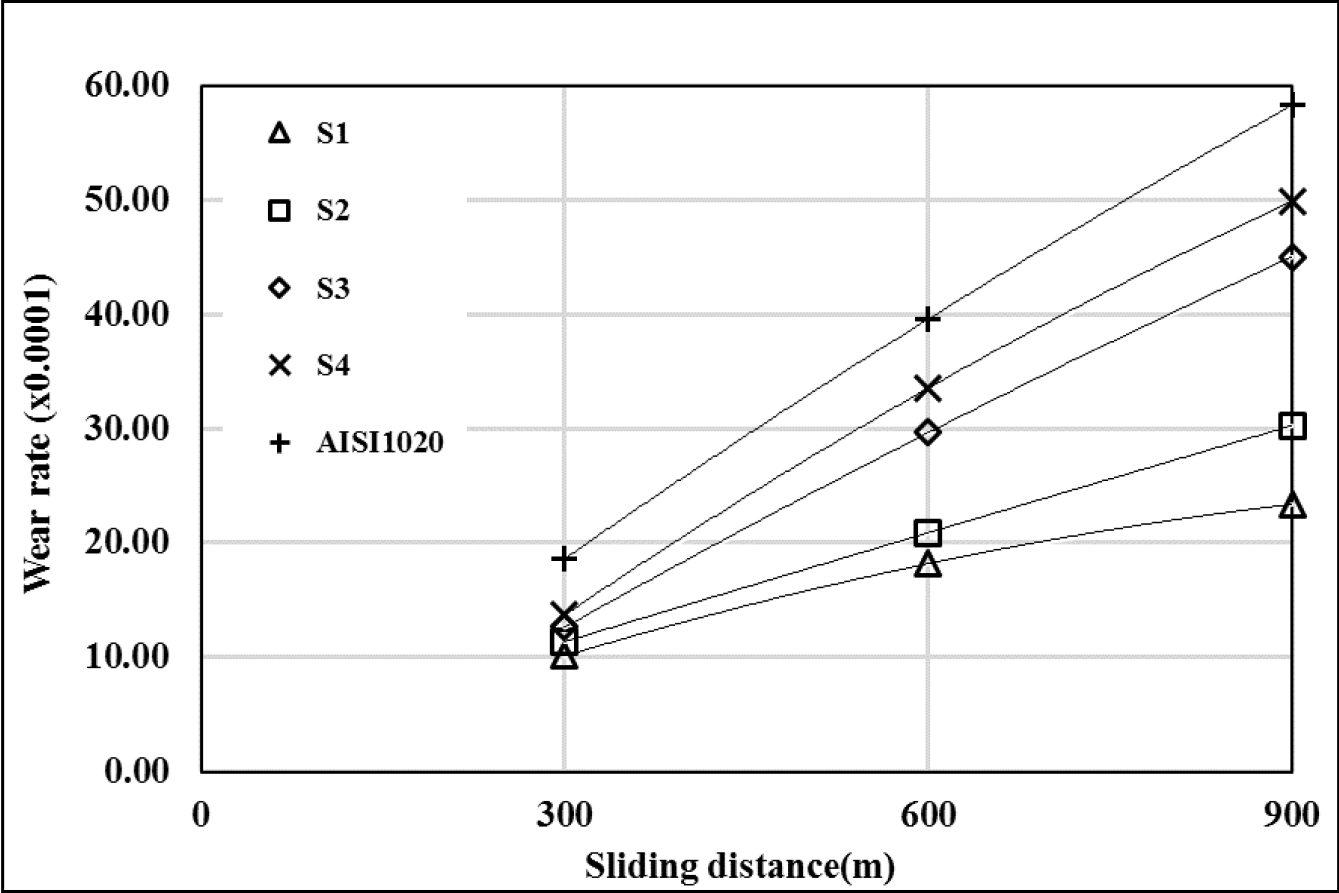
Wear rates at 58.86 N load. (The wear rate is found by multiplying the value of x-axis by 0.0001).

The wear rates of the substrate material and surface coated samples at different loads were shown in Figs 7–9. The wear behaviors of all coated samples were different from each other and the wear rates of all samples are lower than AISI 1020 at all load values. Wear resistance was decreased with increasing heat input, due to intensive melting of substrate material. So the ratio of alloying elements (Cr, W and B) were decreased in melting pool and carbide and boride ratios were decreased because of this. With decreasing carbide and boride amounts in coating layer’s, the hardness was decreased. It is known that hardness has good effect on the wear resistance [18]. The wear resistance of AISI 1020 was increased with increasing sliding distance from 600 m to 900 m at 19.62 N load (Fig 7). The samples, which has high hardness (S1 and S2), wear resistances were increased with increasing sliding distance from 300 to 900 m. Other samples (S3 and S4) wear resistances were increased first and decreased with increasing sliding distance from 600 to 900 m (Fig 7). At 39.24 N load, the wear resistances of AISI 1020 and coated samples were decreased with increasing sliding distance (Fig 8). At 58.86 N load, S1’s and S2’s wear resistances were increased with increasing sliding distance. Also AISI 1020’s, S1’s and S2’s wear resistances were decreased first and then increased with increasing sliding distance from 600 to 900 m (Fig 9). At all load values sample S1, which is coated with lowest heat input, has highest wear resistance. It can be explained by the highest hardness of S1 and the higher ratio of hard boride and carbide phases than the other samples. The sample S4 has the lowest wear resistance which is the most soft sample due to the intensive melting of AISI 1020.

**Fig 10:**
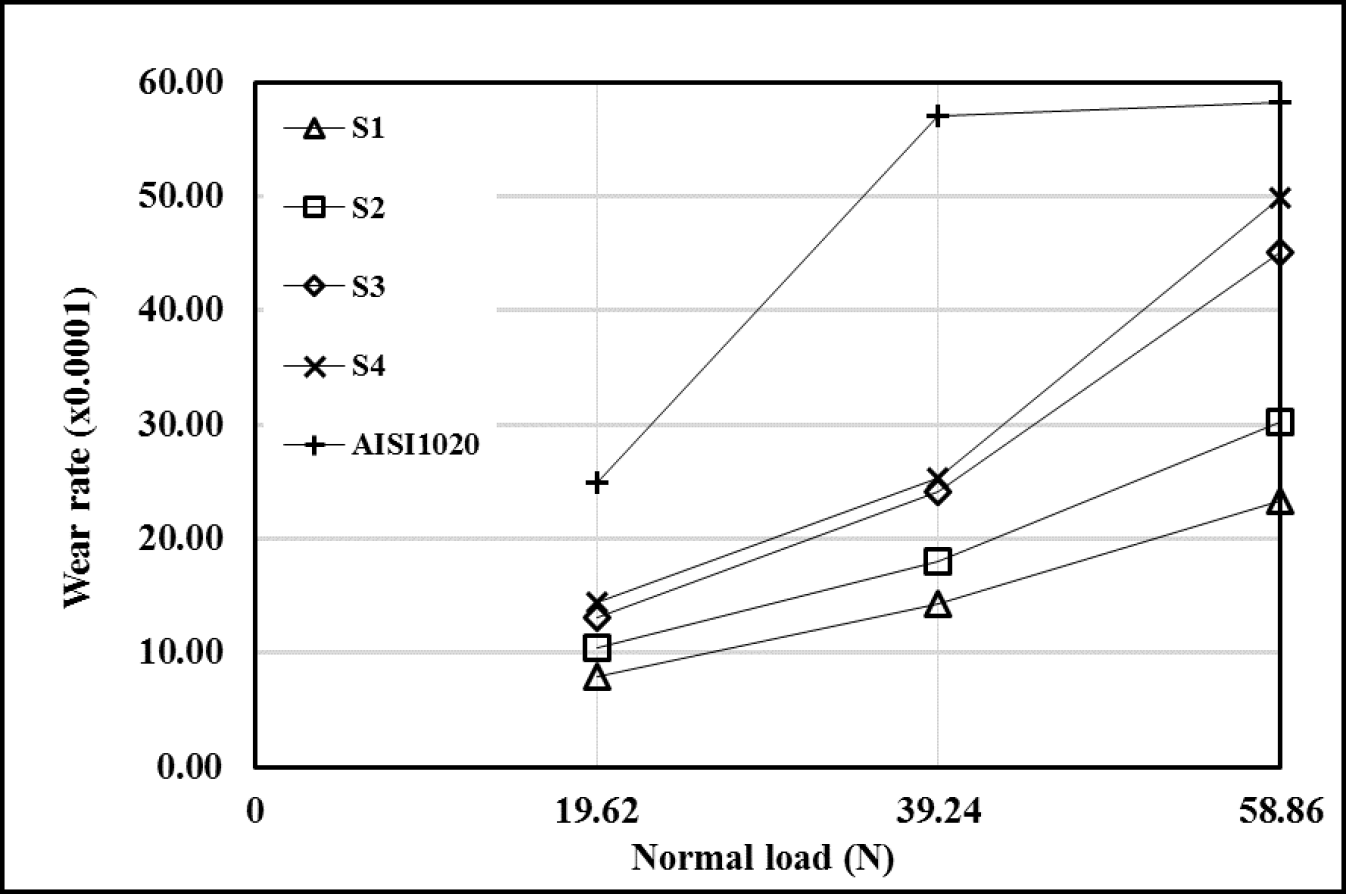
Wear rates according to normal load. (The wear rate is found by multiplying the value of x-axis by 0.0001).

The change in wear rates according to the normal load was seen in Fig 10. The wear resistance of the AISI 1020 decreased when the load increased to 39.24 N while it increased when the load increased to 58.86 N. It can be said that this is caused by the strain hardening and increasing temperature occurred in the substrate material’s sliding surface due to friction [21, 22]. The wear resistances of all coated samples increased when the load increased to 39.24 N and decreased when the load increased to 58.86 N.

**Fig 11:**
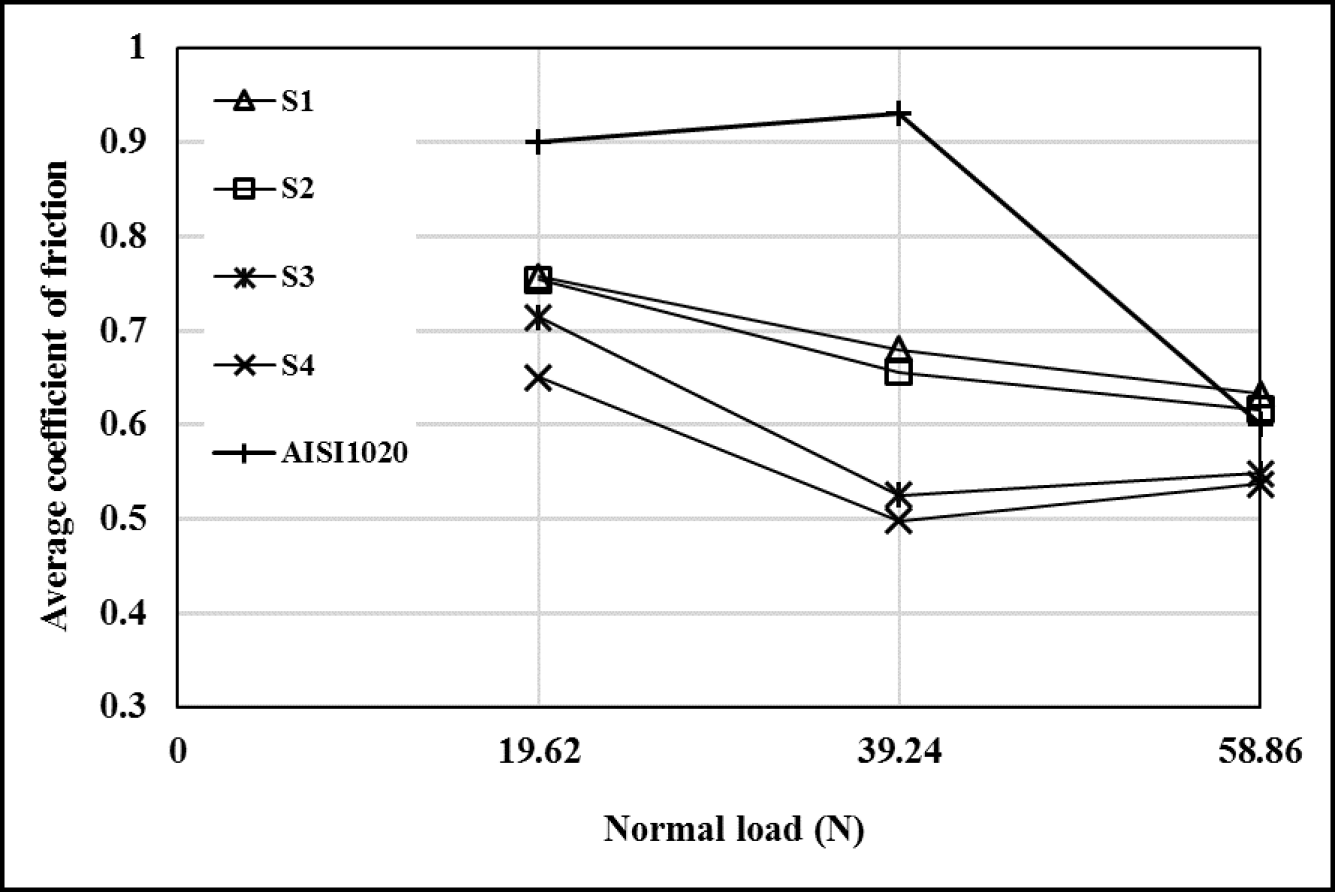
The variation of average coefficient of frictions according to load.

The variation of average friction coefficients (μ_avg._) according to the load was shown in Fig 11. The μ_av._ values of the coated samples and AISI 1020 vary with the change of the load. It has also been pointed out in the previous studies that the average coefficient of friction varies depending on the load [23]. The samples with higher average microhardness have higher μ_avg._ values and as the heat input increases the μ_avg._ decreased. At 19.62 and 39.24 N load, the μ_avg._ values of all coated samples are lower than that of substrate. The average friction coefficients of samples coated with high heat input (S1 and S2) compared to other specimens at 58.86 N load are lower than that of AISI 1020. The μ_avg._ values of the coated samples vary between 0.650–0.757 at 19.62 N load, 0.498–0.680 at 39.24 N load and 0.537–0.633 at 58.86 N load. Also the μ_avg._ values of the AISI 1020 is 0.9 at 19.62 N load, 0.93 at 39.24 N load and 0.6 at 58.86 N load.

**Fig 12:**
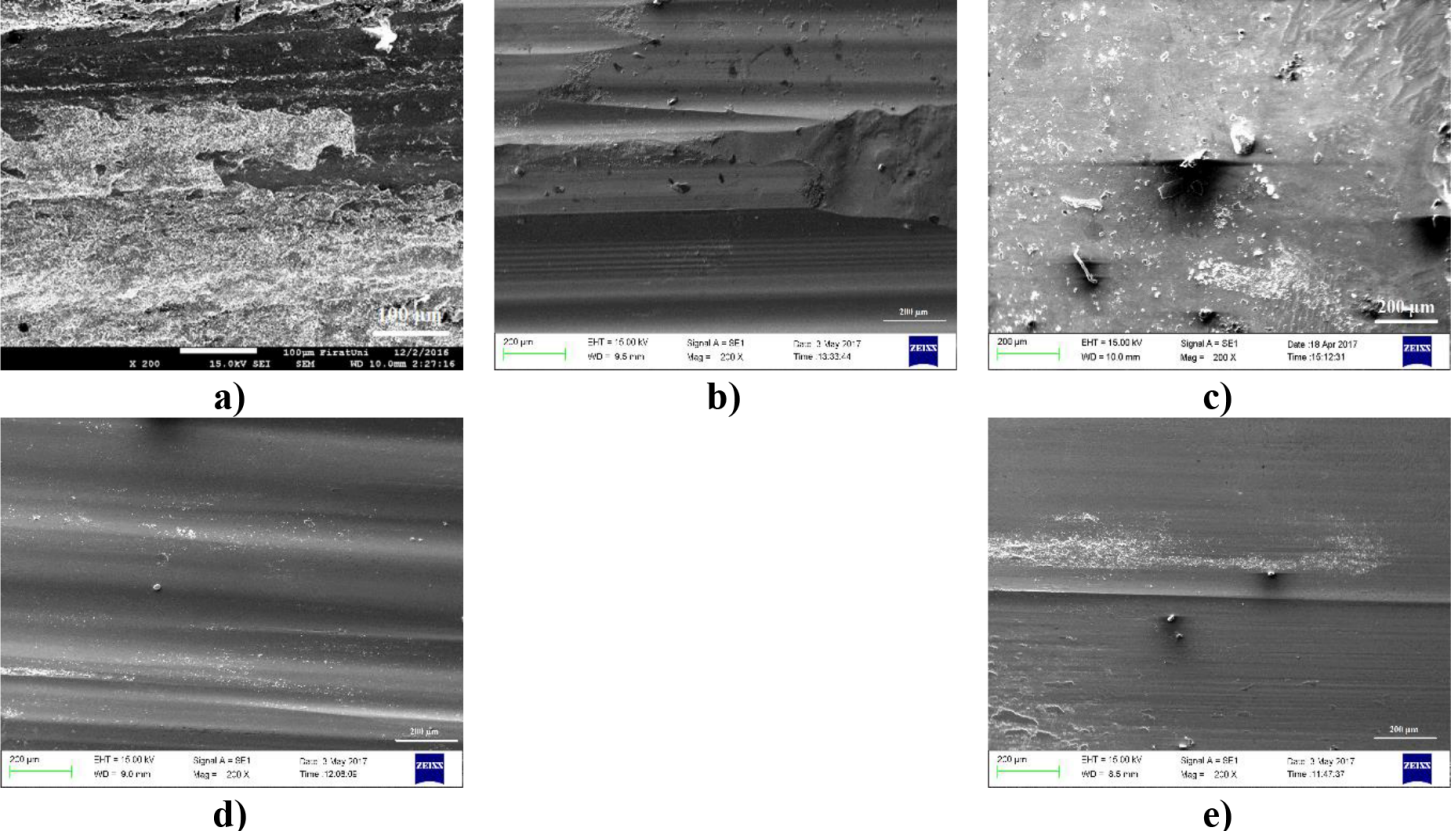
**SEM photographs of the wear surface at 58.86 N load.** a) AISI 1020, b) S1, c) S2), d) S3 and e) S4.

The wear surface SEM photographs of the coated samples and substrate worn at high load (58.86 N) were seen in Fig 12. Significant amounts of the material were lost from AISI 1020 Fig 12A. Wide and deep craters were observed on the worn surfaces of AISI 1020. It was determined that the particles broken from the sliding surface were sticked to the grooves formed on the surface at 58.86 N load due to the high temperature. When the wear surface photographs of the coated samples were examined (Fig 12B–12E), it was observed that the worn surfaces consisted of mostly grooves and micro scratching. Spalling formed on worn surfaces somewhere and the grooves flattened with increasing wear (Fig 12E).

## Conclusions

- The surface of AISI 1020 was successfully modified with Fe-Cr-W-B-C elements by using PTA welding method. No porosity or cracks were encountered on the coated surfaces and interlayers.
- Coating layers depths and interface regions heights increased with increasing heat input.
- The coating layers mainly composed of M_23_(C, B)_6_ (M = Cr, Fe, W) carbide, M_7_C_3_ (M = Cr, Fe, W) carbide, (Cr, Fe)B boride, FeB boride and FeCr.
- Coating layers average microhardness were decreased with increasing heat input and maximum average microhardness was measured to be 1217 HV.
- The wear rates of the coated samples were lower than that of AISI 1020 steel and wear rates of the coating layers increased as their average microhardness decreased.
- The samples with higher average microhardness have higher friction coefficient and while heat input increased, friction coefficient decreased.
